# Podiatrist-Delivered Health Coaching to Facilitate the Use of a Smart Insole to Support Foot Health Monitoring in People with Diabetes-Related Peripheral Neuropathy

**DOI:** 10.3390/s21123984

**Published:** 2021-06-09

**Authors:** Emma M. Macdonald, Byron M. Perrin, Leanne Cleeland, Michael I. C. Kingsley

**Affiliations:** 1 Holsworth Research Initiative, La Trobe Rural Health School, College of Science, Health and Engineering, La Trobe University, Bendigo 3550, Australia; emma.macdonald@gvhealth.org.au (E.M.M.); b.perrin@latrobe.edu.au (B.M.P.); 2 Diabetes Centre, Goulburn Valley Health, Shepparton 3630, Australia; 3 Quality, Risk and Innovation Unit, Goulburn Valley Health, Shepparton 3630, Australia; leanne.cleeland@gvhealth.org.au; 4 Department of Exercise Sciences, University of Auckland, Auckland 1023, New Zealand

**Keywords:** diabetes, health coaching, technology adoption, foot monitoring, telehealth, peripheral neuropathy, diabetes foot disease

## Abstract

This trial evaluated the feasibility of podiatrist-led health coaching (HC) to facilitate smart-insole adoption and foot monitoring in adults with diabetes-related neuropathy. Adults aged 69.9 ± 5.6 years with diabetes for 13.7 ± 10.3 years participated in this 4-week explanatory sequential mixed-methods intervention. An HC training package was delivered to podiatrists, who used HC to issue a smart insole to support foot monitoring. Insole usage data monitored adoption. Changes in participant understanding of neuropathy, foot care behaviours, and intention to adopt the smart insole were measured. Focus group and in-depth interviews explored quantitative data. Initial HC appointments took a mean of 43.8 ± 8.8 min. HC fidelity was strong for empathy/rapport and knowledge provision but weak for assessing motivational elements. Mean smart-insole wear was 12.53 ± 3.46 h/day with 71.2 ± 13.9% alerts not effectively off-loaded, with no significant effect for time on usage F(3,6) = 1.194 (*p* = 0.389) or alert responses F(3,6) = 0.272 (*p* = 0.843). Improvements in post-trial questionnaire mean scores and focus group responses indicate podiatrist-led HC improved participants’ understanding of neuropathy and implementation of footcare practices. Podiatrist-led HC is feasible, supporting smart-insole adoption and foot monitoring as evidenced by wear time, and improvements in self-reported footcare practices. However, podiatrists require additional feedback to better consolidate some unfamiliar health coaching skills. ACTRN12618002053202.

## 1. Introduction

Despite ongoing global efforts to improve foot health outcomes for people with diabetes using a range of strategies [1,2], increasing numbers of people continue to develop preventable diabetes-related foot ulcerations and experience lower limb amputations [1,3,4]. While daily foot checks and regular attendance to primary clinicians for foot monitoring and care are beneficial in maintaining good foot health [5], people with diabetes-related peripheral neuropathy remain at constant risk of developing foot ulcerations due to an inability to detect noxious stimuli, such as the effect of high peak foot pressures, sustained lower peak pressures, or shear [2,6,7]. To augment current preventative foot care regimes, such as orthopaedic footwear and offloading [2], wearable smart foot monitoring devices, including smart socks [8,9,10] and smart sensory insoles [11,12,13,14], are being developed to provide ongoing monitoring of a number of variables in order to provide real-time alerts to the user to undertake protective action in order to prevent injury [8,12,15]. Since the underlying nerve damage caused by diabetes is life-long, people with peripheral neuropathy would need to adopt and use these technologies over many years in order for them to significantly impact foot ulcer incidence. However, patient adherence to currently prescribed footwear and off-loading for ulcer prevention is often poor [2,16]. It is therefore likely that usage of technologies, such as smart insoles that are worn within existing footwear, will also face barriers to adoption, over and above those that exist for footwear, due to a range of psychosocial factors impacting on adoption of diabetes management technologies [17].

Research investigating factors impacting adoption of diabetes management technologies have found many factors interact to affect adoption, such as individuals’ expectation of health benefits from the technology, accessibility, ease of use, social influence from family members, and support from clinicians [17]. While many of these factors are outside the control of clinicians, there is evidence that clinicians can impact patient technology utilisation by the degree they encourage patient use of, and engagement with, the technology and data generated [17]. Two recent Australian studies found generally positive attitudes of regional adults with diabetes and Australian podiatrists towards adopting wearable foot monitoring technology [18,19]. However, both groups had unanswered questions related to device performance and ease of use, and podiatrists had concerns regarding patient-related factors, such as age and footwear usage as well as the clinical time required to support patient smart insole adoption [18,19]. There is scope for podiatrists to play an important role in harnessing their patients’ openness to foot health monitoring technologies to support adoption and utilisation [17]. However, further research is required to optimise podiatrist and patient interactions in order to translate positive adoption intentions into actual adoption.

Health coaching is one approach to enhancing patient motivation and self-efficacy to change and has shown utility in supporting behaviour change in older people [20] and those with chronic illness, such as diabetes [21]. The term ‘health coaching’ has been applied to highly variable interventions, which has made it difficult to define the term [22,23]. Wolever et al. [22] defined multiple elements that comprise health and wellness coaching, including that it is patient centred and that the health professional providing the coaching utilises techniques to support the development of patients’ intrinsic motivation. Health coaching uses a range of techniques to assess a person’s readiness to change, identify health goals and the specific steps and actions required to support achievement of these goals [22,24,25,26,27].

In the context of foot care and foot ulcer prevention for people with diabetes, health coaching could be used to build participant knowledge and address misconceptions about neuropathy and foot care behaviour, encourage the identification of likely outcomes of various behaviours in the presence of neuropathy, and promote the exploration of the personal importance attached to these outcomes [22,23,24,25,27]. This process could build participant self-efficacy in performing foot monitoring and overcoming identified environmental and personal barriers to undertaking protective behaviours [22,24,25,26,27].

The aim of this study was to determine if a health coaching approach to issuing a smart insole with the purpose to augment foot health monitoring is feasible in podiatry practices. Secondary aims were to evaluate changes in patient usage of the smart insole, understanding of neuropathy, and self-reported foot care practices. We hypothesised that:

1. A targeted 120-min face-to-face health coaching training session would be sufficient to enable podiatrists to appropriately use health coaching techniques in clinical consultations with participants to support foot monitoring as measured by a health coaching fidelity assessment tool and qualitative data;
2. Participants would wear and respond to the smart insole alerts throughout the 28-day trial as measured by prospectively gathered smart insole usage data;
3. Participant interpretation of neuropathy and self-reported foot care practices would improve following the health coaching intervention as measured by changes in questionnaire scores and qualitative data; and
4. Trialling the smart insole would influence podiatrists’ and participants’ behavioural intention to use the smart insole in the future as measured by changes in modified Unified Theory of Acceptance and Use of Technology questionnaire domain scores and qualitative data.

## 2. Materials and Methods

### 2.1. Study Design, Settings, and Recruitment

**Figure 1:**
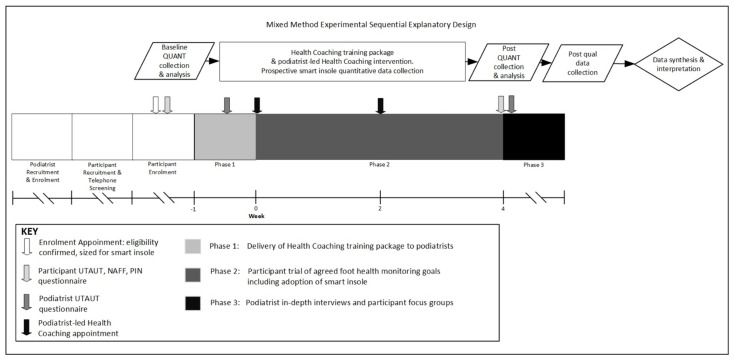
Study design, procedure and timeline.

A quantitative dominant (QUANT-qual) mixed-methods intervention with an explanatory sequential core design was utilised (Figure 1) [28]. Ethics approval was granted by Goulburn Valley Health Human Research Ethics Committee (GVH-2019-171432(v2)) 28 June 2019 and La Trobe University Human Research Ethics Committees (HEC19148) 7 June 2019. The trial was registered with the Australian New Zealand Clinical Trials Registry ACTRN12618002053202. Figure 1 illustrates the study design, procedure, and timeline. The interventional components occurred over a 5-week period during phases 1 and 2, with phase 3 conducted in the month following the intervention.

The trial was located in Shepparton, a regional city in north-central Victoria, Australia. The trial was conducted in one public podiatry practice treating people with high-risk feet and one private practice in order to determine the acceptability of a health coaching approach in these two settings.

Two podiatrists, one practicing in a regional public health setting and the second in a private practice setting, were recruited by email invitation as a convenience sample to participate. The inclusion criteria for podiatrists and participants are provided in Table 1. Both podiatrists invited to participate enrolled and completed the trial as podiatrist health coaches.

Participants were recruited by flyers displayed in the waiting areas and podiatry treatment rooms of each of the participating practices. Volunteers contacted a researcher via telephone to obtain further information about the trial and undergo initial telephone screening to determine eligibility. If eligible volunteers were interested, they were booked for a face-to-face screening appointment and underwent an informed consent process, foot assessment, and baseline data collection. Figure 1 outlines the procedures utilised for this study, including the time points, the order in which data collection tools were administered, and the timing of interventions.

### 2.2. Tools for Data Collection

A tool was developed by the researchers to assess podiatrists’ fidelity to the health coaching training they received in phase 1, and then were to apply during the health coaching appointments at the start of phase 2. Details of the health coaching training package, associated podiatrist and participant resources, and health coaching fidelity assessment tool can be found in Table 2 and Table 3.

The foot monitoring technology selected for use in this study was the SurroSense Rx* smart insole manufactured by Orpyx Medical Technologies (Calgary, AB, Canada). The device prospectively collected data on wear time, number of alerts, and alert response. A description of the device characteristics can be found in Table 2.

Three questionnaires were used pre and post phase 2 in this study: the Nottingham Assessment of Functional Footcare (NAFF) questionnaire [31], the Patient Interpretation of Neuropathy questionnaire [32], and a modified version of the Unified Theory of Acceptance and Use of Technology (UTAUT) questionnaire [33,34]. Details of the tools and their use in the study are provided in Table 2.

### 2.3. Health Coaching Training Package

The 120-min health coaching training package was developed by two researchers, one of whom was a podiatrist and the other a registered nurse with a health coaching background. The training package was delivered to participating podiatrists in phase 1 (Figure 1). The elements of the health coaching training package and podiatrist and participant health coaching resources are outlined in Table 3.

### 2.4. Podiatrist-Led Health Coaching to Support Foot Health Monitoring

The initial health coaching appointments in phase 2 were expected to take 45 min and were audio-recorded on a password-protected smart phone and delivered to the trial’s health coach on a USB for later assessment of each podiatrists’ fidelity to the health coaching approach. During the appointments, podiatrists worked with participants to identify individual foot health monitoring goals, a component of which included the issue of the smart insole for use over the four-week trial. The strategies and tools podiatrists were to use during the health coaching intervention with participants are outlined in Table 3. The participants attended a follow-up appointment a fortnight later. The duration of the review consultations was noted by the podiatrists, but these were not audio recorded.

### 2.5. Quantitative Data Collection and Analysis

The granular raw data from each participant’s SurroSense Rx*smart watch, obtained from Orpyx Medical Technologies (Calgary, AB, Canada), was analysed using a program designed in LabVIEW (version 16; National Instruments, Austin, TX, USA). The researchers used the same definitions for the triggering of an alert as utilised by Abbott et al. [11]. An alert was successfully responded to if pressure on the affected part of the foot was off-loaded within 3 min of the alert being generated. The alert would remain active until pressure was reduced on the area, with the participant receiving reminders to off-load every 3 min. Days where insole wear time was recorded as less than 60 min due to device malfunctions were excluded from analyses. In total, 7 out of a possible total of 280 wear days’ data (2.5%) were excluded from analysis due to technology malfunctions.

Malfunctions included disconnection of leads from the transmission pod, and disconnection between the insole and smart watch. Days where participants had not attempted to wear the device were still included in the analyses. For two participants who had experienced technological malfunctions who chose to continue to wear the device beyond day 28, the additional valid wear days were included in the analyses in substitution for the days excluded due to technology issues.

Descriptive statistics were used for demographic, questionnaire, and insole usage data. Ratio data were presented as mean and standard deviation, and nominal data were presented as proportions. Statistical significance for analyses was set at *p* ≤ 0.05. A trend towards significance was set at *p* < 0.10. All statistical analyses were performed using IBM SPSS Statistics for Windows (version 26.0; IBM Corporation, Armonk, NY, USA). Mean domain scores measured on a continuous 5-point Likert scale for the UTAUT and PIN questionnaire were calculated, and total scores for the NAFF questionnaire and health coaching fidelity tool were calculated. Insole usage data were presented as mean hours worn per valid wear day, mean daily number of alerts, and mean percentage of alerts not successfully responded to. Paired sample t-tests were used to assess changes in pre and post mean domain PIN and UTAUT scores.

Mixed-model analyses of variance were conducted to assess the impact of practice setting (between) and time (within) on weekly insole wear, number of alerts, number of alerts not responded to, percentage of alerts not successfully responded to, and the self-reported foot care behaviours of participants as measured by the NAFF over the four-week trial. Significant interaction effects were interpreted to indicate that the practice setting influenced the pattern of response over time. Where the interaction effect did not reach significance, the main effects of practice setting and time were consulted. Significant timing effects were further explored using Bonferroni–Holm adjusted pairwise comparisons.

### 2.6. Qualitative Data Collection

In phase 3, focus group and in-depth interviews were conducted to enable participants to provide context to, and exploration of, the quantitative data and the semi-open-ended UTAUT responses [28]. In particular, it was an opportunity for participants and podiatrists to relate their experiences of the health coaching approach taken to foot monitoring and the process of adopting a smart insole. Focus group questions included ‘Did the (health coaching) consultations with your podiatrist impact your understanding of nerve damage in your feet and how to look after your feet?’, ‘Did the written information provided to you, including the instruction manual, Quick Start Guide, laminated response to alerts pictograph and the action plan you completed enable you to confidently use the smart insole at home, resolve alerts or other issues?’ In-depth interview questions included ‘Was the health coaching training package understandable, and did it go into sufficient depth?’, ‘How did the health coaching skills impact your communication with your client and the provision of foot care/monitoring information, including the device issuing?’ Focus group and in-depth interviews were audio recorded using VoiceMemo on an i-phone 8 password-protected phone and then transcribed verbatim and analysed thematically using NVivo version 12.6.0 3841 (QRS International Pty Ltd., Chadstone, Australia). All coding was completed independently by two authors, with codes then being discussed to develop themes. Focus group and in-depth interview data related to technology adoption utilised thematic coding derived from the UTAUT domains. All other data was coded to describe the meaning of the text, then grouped into categories and then themes that could explain the quantitative data [36].

While it was anticipated that all participants would attend a focus group, 7 out of 10 attended on the day. Four out of five attended the private arm focus group; one woman aged 71 years with type 2 diabetes for 19 years, and four men aged between 68 to 79 years all with type 2 diabetes for between 10 to 18 years. Three out of five public arm participants attended their scheduled focus group. One failed to attend due to being out of the region and one could not be contacted to confirm the date and time of the session. One woman aged 58 years with type 1 diabetes for 34 years, and two men aged between 72 and 74 years both with type 2 diabetes for between 10 to 23 years attended the public arm focus group. Both podiatrists attended an in-depth interview, one male aged 32 years employed in public practice with 8 years professional practice and one male aged 58 years from private practice with 37 years of professional practice.

## 3. Results

The podiatrist-led health coaching appointments took a mean of 43.8 ± 8.8 min for the initial appointment and 29.6 ± 12.9 min for the review (Table 4). Participants used the smart insole for a mean of 12.53 ± 3.46 h per day and received a mean of 22.96 ± 12.9 daily alerts during the trial, of which they failed to effectively offload a mean of 71.2 ± 13.9 percent of alerts within 3 min of being generated (Table 4).

Table 5 provides the results for the health coaching fidelity tool. The mean health coaching fidelity score was 30.8 ± 2.0 out of a maximum possible total of 58. The health coaching fidelity tool revealed that podiatrists’ use of health coaching techniques related to motivational elements, including assessing participants’ readiness to adopt a smart insole and practice protective foot care behaviours (HC 4), the importance they attached to smart insole adoption and protective footcare behaviours (HC 5, 12, 13), and their confidence in their ability to do this (HC 14, 15), were either not used or only partially used (Table 5). While elements of the intervention related to being participant centred (HC 2, 3, 10, 11) demonstrated higher mean values than the motivational elements, their use was still inconsistent. Podiatrists most consistently used elements of the intervention related to providing knowledge to participants on how to use the smart insole, the nature of neuropathy and protective footcare behaviours (HC 6, 7, 8, 9, 16), and those related to demonstrating empathy and building rapport (HC 1, 17).

Practice setting did not influence the pattern of response over time for the variables mean daily hours per week of insole wear, mean daily number of alerts per week, mean daily number of alert non-responses per week, mean daily percentage per week of alert non-responses, and NAFF scores (interaction effect: *p* ≥ 0.236) (Table 6).

There was no significant main effect for time on mean daily hours of insole wear F_(3,6)_ = 1.194, *p* = 0.389, η^2^ = 0.374 and mean daily percentage of alert non-responses F_(3,6)_ = 0.272, *p* = 0.843, η^2^ = 0.12, which remained consistent over the four weeks of the trial. However there was a significant main effect for time on the mean daily alerts per week, F_(3,6)_ = 4.92, *p* = 0.047, η^2^ = 0.711, and mean daily alert non-responses per week F_(3,6)_ = 5.38, *p* = 0.035, η^2^ = 0.738, which significantly reduced from week 1 to week 4, *p* = 0.043. There was also a trend towards a significant change over time on NAFF total score F_(1,8)_ = 4.29, *p* = 0.072, η^2^ = 0.349, with both groups increasing their mean total NAFF score compared to baseline (Table 6).

Practice setting did not significantly affect insole usage or response during the four-week trial, with no significant main effects for mean daily insole wear F_(1,8)_ = 0.135, *p* = 0.723, η^2^ = 0.017, mean daily alerts F_(1,8)_ = 0.665, *p* = 0.438, η^2^ = 0.077, mean daily alert non-responses F_(1,8)_ = 1.136, *p* = 0.318, η^2^ = 0.124, and mean daily percentage of alert non-responses F_(1,8)_ = 0.162, *p* = 0.698, η^2^ = 0.020. However, there was a trend toward significance for the main effect of practice setting and changes in NAFF total score F_(1,8)_ = 5.012, *p* = 0.056, η^2^ = 0.385, with private participants having lower mean pre and post NAFF scores than public participants (Table 6).

UTUAT scores related to future adoption intentions of the smart insole demonstrated significant post trial reductions in mean participant attitude t = 2.6, *p* = 0.028, and behavioural intention t = 3.4, *p* = 0.008. There was also a trend towards a significant reduction in participants’ performance expectancy t = 2.15, *p* = 0.060 and an increase in self-efficacy t = −1.96, *p* = 0.081 from baseline to post trial (Table 7). Changes in pre and post PIN domain mean scores demonstrated a significant improvement in domains evaluating participants’ understanding of the causes of neuropathy and foot ulcers (item C1) t = −2.74, *p* = 0.023, foot ulcer onset (item TL) t = −2.70, *p* = 0.024, as well as allocation of responsibility for developing foot ulcers (item C2) t = −3.03, *p* = 0.014 (Table 7).

### 3.1. Qualitative Results

#### 3.1.1. Health Coaching Intervention

In the in-depth interviews, the podiatrists reported that they felt that the two-hour education session, with the additional written information, was sufficient to be able to coach participants in foot health monitoring and the use of a smart insole. However, the private podiatrist felt that the training would have been enhanced by receiving feedback on their performance following their initial health coaching consultation to assist in consolidating the procedure (Table 8).

The health coaching education prompted the podiatrists to reconsider their approach to communicating with participants, particularly relating to the provision of knowledge and tailoring the session to each individual’s needs. In particular, podiatrists modified their usual practice by utilising health coaching tools, such as Teach Back, to confirm participant understanding in order to correct misunderstandings and fill knowledge gaps. For the private podiatrist, this process also enhanced and improved his relationship with participants (Table 8).

Participants in the private focus group reported that the way information was delivered by the podiatrist gave them far greater insights into their condition and how to care for their feet than previous education, and that the information was more personal and relevant to them (Table 9). Participants in the public focus group felt that the podiatrist made themselves ‘available’ throughout the consultation, and that his delivery of foot care knowledge and overall execution of the health coaching sessions was effective. However, they felt that the information provided regarding the nature of neuropathy and recommended protective foot care behaviours confirmed their existing understanding rather than providing them with new information (Table 9). Participants in both focus groups reported that the health coaching sessions and written information provided them with sufficient knowledge and confidence to monitor their feet and adopt the smart insoles successfully (Table 9).

#### 3.1.2. Performance Expectancy

Participants and podiatrists could see the potential benefits of foot monitoring technologies, like the smart insole, in providing greater knowledge about foot pressure areas. However, they felt that further development of the device utilised in this trial would make it more user friendly (Table 8 and Table 9). Some participants in the private arm were perplexed by the alerts they received and how the device defined ‘high pressure’, particularly in the context of being seated. For some private arm participants, the feedback appeared random and significantly diminished their level of trust in the feedback they received (Table 9). However, participants in the public arm who had previously experienced foot ulcerations understood why they were being alerted to pressure in a specific foot location and believed the device was providing accurate feedback but felt that there was little they could do to permanently address the pressure issues due to underlying structural foot deformities (Table 9).

#### 3.1.3. Effort Expectancy

Participants found some elements of the device, such as charging the batteries and connecting the innersoles to the smart watch, to be easy when everything functioned as intended. However, participants experienced frustration or a sense of added burden when elements malfunctioned or when they received repeated alerts that became intrusive during daily tasks, such as driving or preparing meals. Public arm participants’ comments indicate that they found using the smart insoles less burdensome than participants in the private arm (Table 9).

#### 3.1.4. Social Influence

Socially, some participants found that the audible alarms were disruptive when they were socialising with others, reporting that they were questioned about the alarms and the presence of transmission pods, which some found off-putting (Table 9).

Podiatrists’ attitudes towards the smart insole were strongly influenced by their patients’ (participants) experiences and opinions of the device, in particular the private podiatrist. Poor participant experiences with the smart insole exerted social influence that negatively impacted podiatrists’ intentions towards adopting the device into practice (Table 8).

#### 3.1.5. Facilitating Conditions

Participants reported that they had the resources required to successfully use the smart insole. However, the restrictiveness of having to wear lace-up or Velcro-enclosed footwear to use the device was seen as a deterrent for those who preferred flexibility in the type of footwear that they wore through the day, particularly when at home (Table 9).

Podiatrists believed the health coaching approach to supporting foot health monitoring, including the issuing and education in the use of a smart insole, was suitable in both public and private settings in terms of the length of consultation times and practice resources required (Table 8).

#### 3.1.6. Behavioural Intention

While participants and podiatrists were confident in their capacity to use the smart insole and could see potential benefits to the device, particularly for those who had experienced previous foot ulcers, neither group intended to adopt this version of a smart insole in the future. However, both participants and podiatrists believed that with further development, foot monitoring technologies could play a valuable role in preventing foot ulcerations, and they would be open to exploring future iterations of a smart insole (Table 8 and Table 9).

## 4. Discussion

The use of health coaching techniques to support foot health monitoring is feasible in both private and public podiatry practice settings, as evidenced by the mean duration of heath coaching appointments and focus group and in-depth interview responses. Podiatrists using health coaching techniques supported participants’ foot monitoring and adoption of a smart insole over a four-week period, as evidenced by insole usage data, improvements in total NAFF scores and individual PIN domains, and confirmed by focus group responses. However, the health coaching fidelity scores indicate that despite the education podiatrists received in using a range of health coaching strategies and the importance of being participant centred, podiatrists at times directed the consultations, rather than being primarily participant directed. Podiatrists were most comfortable providing education, building rapport, and demonstrating empathy to participants, skills that they were familiar with utilising in usual clinical practice rather than fully embracing more unfamiliar health coaching techniques.

Low health coaching fidelity scores in the domains related to improving participant motivation and moving through the stages of change could have been due to the fact that participants in this study were already motivated, as evidenced by the high baseline UTAUT behavioural intention scores toward smart insole adoption. Therefore, the podiatrists might have made assumptions about participant motivation being high for all aspects of foot health monitoring rather than explicitly confirming each element. However, once podiatrists had confirmed participant readiness to undertake change actions, it would have then been appropriate to move beyond the motivational elements [27]. The 120-min training package without further reinforcement was insufficient to consolidate unfamiliar health coaching skills to podiatrists’ practice. As one podiatrist stated in the in-depth interview, consolidating these skills would have been helped by receiving feedback about the quality of their health coaching technique immediately following their first appointment. Receiving ongoing feedback to consolidate coaching skills has been shown to improve fidelity in other targeted behaviour change interventions [37]. In future, an opportunity for more feedback should be incorporated into this type of targeted health coaching intervention to further support consolidation of trainee skills in order to improve their capacity to support participant self-determination [27,37].

The mean of 12.5 ± 3.5 h of smart insole and footwear daily use indicates that participants successfully adopted the insole and complied with directions to wear it consistently throughout the study, exceeding the 60% and approaching the 80% of the day threshold for shoe wear suggested to reduce ulceration recurrence [7,38]. This result was considerably higher than previous studies using a similar device without health coaching to support adoption, but it must be noted the other trials were for longer durations. A three-month trial had a mean of 5.4 ± 3.4 h per day for smart insole use [12], and an 18-month RCT reported a median of 6.1 h (range 4.3–7.6) of daily wear time [11]. However, while participants in this study were diligent in their daily wear, there was a relatively low percentage of alerts that were effectively off-loaded within the prescribed three-minute time frame, even with a significant decline in alert frequency over the four weeks. Najafi et al. [12] reported that their high-alert group, which they defined as receiving a mean of 0.75 alerts per hour, improved their successful response to alerts over time compared to their low-alert group but posited that there was likely an upper threshold at which additional alerts would lead to declining adherence. It is likely, based on focus group responses, that participants in this trial, who received a mean of 1.83 alerts per hour of wear, developed response fatigue, contributing to the low percentage of successful responses.

Despite the inconsistent application of motivational elements of the intervention, the focus group and in-depth interview responses indicate that the health coaching approach did improve communication with participants and, for the private practice participants, comprehension and internalisation of the nature of neuropathy and the personal implications for their foot health. The impact of this internalisation of foot health appears to be borne out by changes in the NAFF total score and some PIN domains following the intervention. The increase in NAFF scores reflects self-reported improvements in protective foot care behaviours by participants, which might reflect that podiatrists were successful in supporting participants’ sense of autonomy and competence in foot health monitoring, a key goal of coaching [22,27]. However, improvements in these scores could also have occurred simply as a biproduct of participation in the trial leading to a greater awareness of the need to monitor their feet during the study.

Participant dissatisfaction with elements of insole usage were reflected in the significant reduction in post mean participant attitude and behavioural intention towards the smart insole. These results indicate that the lived experience of using the insole negatively impacted participants’ perceptions of the device. This finding contrasted with Najafi et al. [12], who found that participants who received significantly fewer alerts per hour compared to the current study had positive adoption intentions towards a similar device at the end of a three-month trial.

The trend towards a significant reduction in mean UTAUT performance expectancy from baseline to post trial when considered in the context of the focus group and in-depth interview responses indicates that the performance, or functionality, of the device used in this study did not meet participants’ or podiatrists’ original expectations. Previous research in a similar patient population investigating factors that impacted intention to adopt a smart insole found that performance expectancy moderated attitude, which was a predictor of adoption intention [18]. It is therefore unsurprising that the reduction in performance expectancy and attitude negatively impacted our participants’ future adoption intentions. Another study investigating factors impacting Australian podiatrists’ intentions to adopt smart insoles in practice [19] found that performance expectancy was the sole predictor of behavioural intention. Again, it is unsurprising, given a reduction in podiatrists’ performance expectancy mean scores from baseline, that there was also a reduction in podiatrists’ mean post trial behavioural intention score. However, both participants and podiatrists still emphasised that they saw value in real-time foot monitoring and were open to trying future versions of this or other foot monitoring devices.

It is likely that continuing developments in foot monitoring technology would address many of our participants’ concerns and better support adoption and utilisation in the future. For example, the new generation of Orpyx SI Sensory Insole shows promise [39], addressing many of the technological and social issues identified in this study and providing additional desired functionalities. By enabling greater flexibility of alert notifications, the newer generation of insole might address the issue of alert frequency, which was so concerning for our participants, and the additional automation of device functions is likely to reduce the level of effort required for use. The integration of external components within the body of the custom-milled form addresses some of the social concerns identified by our participants related to the visibility of external components and also enables use in wider variety of footwear, a functionality identified in a previous study to be desired by Australian podiatrists [19]. Furthermore, the addition of temperature in conjunction with pressure monitoring is a physiologic parameter the podiatrists in this study identified as being of clinical importance, and which might make it more attractive to Australian podiatrists when considering clinical adoption. Dialogue between device developers and end users is likely to support a process of ongoing innovation and development that will make future foot monitoring technologies more user friendly and attractive to target populations.

Focus group discussions regarding a lack of trust in the alerts by some private practice participants, and the feeling that the insoles alerted when they did not perceive there to be ‘high pressure’ indicates that participants required further exploration of the types of plantar pressures that can lead to tissue damage (peak versus sustained) during their health coaching sessions. Future research using health coaching to support foot monitoring technology adoption should highlight the need to explore participant understanding of the way a device monitors and measures target variables and how the variables relate to their foot ulcer risk. The smart insole used did not report peak pressures but rather pressures >35 mmHg, which were sustained for 95–100% of the time, and if maintained for 15 min or more, generated an alert. Some private practice participants, none of whom had had previous foot ulcerations, struggled to understand how they could have ‘high’ pressure on their feet while sitting down with their feet resting on the floor. A lack of trust in alerts might have also contributed to the relatively low percentage of successful alert responses in this trial. Our participants’ experiences of receiving regular static alerts are consistent with a recent study where a majority of participants reported receiving alerts while in static positions, and resolved alerts with regular bouts of foot movement [11]. While Abbott et al. [11] reported statistically significantly fewer cumulative numbers of foot ulceration sites in their intervention group compared to their control group, they did not find a statistically significant difference between the intervention and control groups in the overall number of people who developed ulceration. While it is encouraging that the intervention group developed fewer numbers of ulcers, podiatrists and participants in our study required compelling evidence that use of a smart insole would significantly decrease the likelihood that they would develop foot ulcers (performance expectancy) in order to support ongoing usage.

## 5. Conclusions

The results of this study demonstrate that it is feasible to use a health coaching approach to issuing a smart insole in the context of foot care health monitoring for adults with diabetes. A strength of health coaching is that the strategies and tools used in this approach, once consolidated by the practitioner, can be applied to behaviour change in a wide range of contexts, including to support different types of technology adoption. However, while a 120-min training session for podiatrists was sufficient for them to successfully issue the smart insole and improve participant internalisation of protective foot practices and knowledge, unfamiliar aspects of the health coaching approach were not sufficiently consolidated. This indicates that further refinement of the package, which incorporates additional trainee feedback, utilisation of fidelity assessment for review appointments, and further development and validation of the fidelity tool, is warranted. After the package and fidelity tool have been refined, they should be tested in a larger prospective trial over a longer duration to assess the influence of health coaching on foot monitoring behaviour change over time, including smart insole wear and alert responses. Additional study limitations are the small sample size, the potential for self-selection bias in those who volunteered to participate compared to those who did not, and that changes in foot care practices were self-reported through questionnaire and focus group responses without objective confirmation.

Despite participants’ successful adoption of the smart insole during the trial, their attitude and behavioural intention towards future adoption were negatively impacted by their experiences. However, focus group and in-depth interview responses indicate that this population remain optimistic about the role of technology in supporting foot monitoring. Participant and podiatrist comments indicate that evidence of device efficacy in preventing foot ulcerations would improve trust, and additional device refinement to improve performance would all increase the likelihood of future adoption. Foot monitoring technology developers should continue to invest in prospective trials to assess device efficacy in preventing foot ulceration and consult with target users regarding device design features.

## Figures and Tables

**Table 1:** Podiatrist and participant inclusion and exclusion criteria.

Inclusion Criteria	Exclusion Criteria
Podiatrists Registered Australian podiatrist. Actively consulting with clients who have diabetes-related peripheral neuropathy. Willing to commit the time required to participate in the trial.	Podiatrists Unwilling to volunteer time to participate in the trial.
Participants Aged > 18 years. Type 1 or Type 2 Diabetes. Willing to wear footwear with a fastening compatible with smart insole. Willing to attend appointments in the regional centre. Diagnosed with diabetes related peripheral neuropathy by impaired detection of 10 g monofilament 128 Hz graduated tuning fork Two-point discrimination [29,30].	Participants Active foot ulceration or infection. Peripheral arterial disease with Absolute Toe Pressure < 60 mmHg. Transmetatarsal or more proximal amputation. Weight over 136 kg as per SurroSense Rx* guidelines. Unwilling to wear compatible footwear.

**Table 2:** Study Tools.

Tool	Description	Trial Usage
Health Coaching Fidelity Assessment Tool	The tool was developed by the researchers for this study. The fidelity elements assessed were aligned to the content of the training package outlined in Table 3 and the underlying transtheoretical model of behaviour change and social cognitive theories which underpinned the components of the skills taught to podiatrists [22,26,27,35]. Content validity was assessed by the health coach who delivered the intervention by comparing the domains of the tool to the health coaching training content. Face validity of the tool was then confirmed by two independent health coaches who provided feedback advising some alterations to phraseology be made. The changes were made, reviewed by the study health coach, and approved prior to finalisation of the tool. Audio recordings of the health coaching appointments were analysed and scored by the study health coach.	Used to assess podiatrists’ fidelity in using health coaching techniques taught in phase 1 with participants in phase 2 consultations.
SurroSense Rx* insole manufacture by Orpyx Medical Technologies (Calgary, AB, Canada).	Each SurroSense Rx* insole utilised 8 pressure sensors distributed to measure plantar foot pressures greater than 35 mmHg, and alert the wearer if pressures greater than 35 mmHg were sustained for 95 to 100% of the time in a 15 min sampling window on the same sensor [11]. Data and alerts were wirelessly transmitted from the transmission pod secured to the top of participants’ footwear to a smart watch worn by participants on the wrist. The smart watch stored data until upload to participants’ Orpyx Connect accounts. Alerts were provided to the wearer via the smart watch as vibration or audible alarms. The smart watch provided information regarding the site on the foot where pressures greater than 35 mmHg had been sustained, and provided instructions on how to off-load the pressure from the affected area. A successful response to an alert was achieved if participants were able to reduce the pressure on the affected area within 3 min of the initial alert. Podiatrists were instructed to calibrate the sensors on the insoles for each participant at the time of issue, and check calibration at the 2-week review appointment.	SurroSense Rx* insole prospectively recorded hours of insole wear, numbers of alerts received by the user, and numbers of successful and unsuccessful responses to alerts during phase 2. These data were used to determine the degree to which participants adopted the smart insoles during the trial.
Nottingham Assessment of Function Footcare (NAFF) Questionnaire	A 29-item validated questionnaire designed to measure self-reported footcare behaviours engaged in by people with diabetes related peripheral neuropathy [33].	Utilised pre and post phase 2. NAFF scores were used to assess association of the health coaching intervention with participants’ self-reported footcare behaviours.
Patient Interpretation of Neuropathy (PIN) Questionnaire	Validated 39 item questionnaire designed to assess the perceptions of peripheral neuropathy of people with diabetes [32]. The tool utilises a 5-point Likert scale ranging from 1 as strongly disagree to 5 as strongly agree. PIN Domains are as follows: ID1: Good circulation equals healthy feet ID2: Accurate interpretation of diabetes related peripheral neuropathy ID3: Foot ulcers would be painful C2: Blame of self or practitioner for peripheral neuropathy and associated consequences. C1: Physical causes of foot ulcers TL: Understanding of ulcer onset CC1: Efficaciousness of foot self-care at preventing consequences of peripheral neuropathy CC2: Degree of control that the practitioner has on foot ulcers Cons: Possible consequences of peripheral neuropathy EC1: Concern about possible consequences of peripheral neuropathy EC2: Anger targeted towards practitioners related to peripheral neuropathy.	Utilised pre and post phase 2. PIN mean domain scores were used to assess association of health coaching intervention with participants’ interpretation of neuropathy.
UTAUT	A version of the Unified Theory of Acceptance and Use of Technology (UTAUT) questionnaire [33]. The modified version of the UTAUT used in this study was validated for use in a health care context with both health providers and patients and contained 29 questions measured on a continuous 5-point Likert scale ranging for 0 as strongly disagree to 4 as strongly agree [34]. The questionnaire measured seven dependent psychosocial variables known to influence technology adoption: performance expectancy, effort expectancy, attitude, social influence, self-efficacy, anxiety, and facilitating conditions, and the independent outcome measure of behavioural intention. In the context of this study, performance expectancy was the degree to which the individual believed that the smart insole would help them to prevent foot ulceration. Effort expectancy was how easy the individual found the smart insole to use in order to monitor their feet. Social influence was the degree to which significant others (e.g., family members, allied health professionals, clients) influenced the adoption of the smart insole. Self-efficacy was the degree to which the individual believed that they had the skills to adopt the smart insole. Facilitating conditions refers to the degree to which the individual believed that they had the capacity and infrastructure required to use the smart insole. Attitude was the individual’s feelings towards using the smart insole. Anxiety was the self-reported degree of anxiety or hesitation the individual experienced in relation to using the smart insole. Behavioural intention was the individual’s intention to use a smart insole over the 4-week period of the trial, and following conclusion of the trial, at some point in the future.	Utilised pre and post phase 2 to measure the impact of smart insole use on psychosocial factors impacting on behavioural intention to adopt the smart insole.

**Table 3:** Health coaching training package and written materials.

120-min Health Coaching Training Package Components	Purpose
Powerpoint presentation	Describe the theoretical underpinnings of the health coaching approach [22,26,35]. Provide an explanation of specific health coaching skills including empathy, active listening, Teach Back, setting S.M.A.R.T goals, and assessment of participant Readiness, Importance, Confidence, and knowledge (R.I.C.k.) [24,25,27] that podiatrists were to use to work with participants during the health coaching intervention. Explain to podiatrists how to work with participants to work through a process of self-discovery [22,26,27] in relation to participant understanding of peripheral neuropathy and how it impacts on their own foot health.
Video recording of a mock structured health coaching appointment	Demonstration of how to apply individual health coaching elements to conduct a health coaching appointment in order to work with participants to identify foot health monitoring goals, and to assess and improve participants’ readiness, importance, confidence and knowledge in setting and achieving their goals, thereby maximising participant motivation, self-efficacy, and self-determination.
Mock health coaching appointment	Podiatrists were asked to demonstrate the health coaching skills learnt from the package to trainers in a role-playing mock appointment where they issued the SurroSense Rx* insole in order to reinforce health coaching skills. Feedback was provided to the podiatrists by the study health coach.
Written materials to support health coaching intervention	
Laminated R.I.C.k. scale	To prompt the podiatrist to check each of these elements related to foot monitoring and smart insole adoption with the participant during consultation.
Specific Measurable Achievable Realistic Timely (S.M.A.R.T.) goals template	To be used by participants to record their individual S.M.A.R.T. goals for foot health monitoring or smart insole use.
SurroSense Rx* user manual Orpyx Industries Pty Ltd.	Used as a reference for both the podiatrist and participant during issue and adoption of the smart sensory insole.
Laminated pictorial instruction guide on resolving SurroSense Rx* insole alerts	Participant quick reference guide to support their learning how to respond to alerts.
SurroSense Rx* Quick Start Guide Orpyx Industries Pty Ltd.	Brief five-step guide to charging, donning, and connecting SurroSense Rx* insole. Used as a quick reference guide for participants when learning how to use the SurroSense Rx* Insole.

**Table 4:** Descriptive statistics *n* = 10.

Variable	Measurement
Sex, Male	7 (70)
Age, Years	69.9 ± 5.6
Years Diagnosed with Diabetes	13.7 ± 10.3
Diabetes Type 2	9 (90)
History of Ulcer	3 (30)
Educational Level (Post-High School Qualification)	6 (60)
Country of Birth, Australia	7 (70)
Technology Use Mobile device with internet	7 (70)
Mean Hours Daily Insole Use	12.53 ± 3.46
Mean Daily Number Alerts	22.96 ± 12.9
Mean Daily Percentage Alerts Not Effectively Off-loaded	71.82 ± 13.51
Initial Consultation Time (min)	43.8 ± 8.8
Review Consultation Time (min)	29.6 ± 12.6
Prior Relationship With Health Coach	6 (60)

Data are presented as number (%) or mean ± SD.

**Table 5:** Health coaching fidelity assessment tool *n* = 10.

Health Coaching Fidelity Domain		Mean (SD)
HC1	Did the HC introduce themselves and set the agenda, ensuring that the consultation content is explained to the participant?	2.6 ± 0.52
HC2	Did the HC introduce the Action Plan and invite participant to use it to set S.M.A.R.T. goals and record information/tasks?	1.9 ± 0.32
HC3	Did the HC encourage participant self-discovery regarding neuropathy and foot protection practices?	1.4 ± 0.52
HC4	Did the HC check readiness to adopt the insole and work on foot health goals?	1.2 ± 0.42
HC5	Did the HC check importance to adopt the insole and work on foot health goals?	1.0 ± 0.00
HC6	Did the HC check knowledge about peripheral neuropathy and foot care practices, while assessing and respecting the participant’s prior knowledge and current actions?	2.80 ± 0.63
HC7	Did the HC provide knowledge on neuropathy and daily foot care practices?	2.67 ± 0.50
HC8	Did the HC provide knowledge on insole usage?	3.00 ± 0.00
HC9	Following education, to what extent did the HC review/reassess participant knowledge and required actions/goals, using techniques, such as Teach Back or having the participant demonstrate tasks etc?	2.40 ± 0.52
HC10	Did the HC help the participant to generate options for taking action within the nominated action area?	1.90 ± 0.57
HC11	Did the HC promote collaboration to set appropriate participant-centred goals with the participant?	1.30 ± 0.48
HC12	Did the HC check importance of nominated goals for both adopting insole and foot health practices?	1.00 ± 0.00
HC13	If importance was < 7, did the HC provide knowledge to build importance?	1.00 ± 0.00
HC14	Did the HC check confidence to undertake nominated actions to achieve agreed goals related to foot health and insole usage?	1.50 ± 0.53
HC15	If confidence was < 7 were concerns discussed and actions simplified?	1.14 ± 0.38
HC16	Did the HC discuss supports and resources available to support participant’s action plan attempts?	2.40 ± 0.52
HC17	Did the HC establish rapport and demonstrate empathy with the participant throughout the consultation?	2.40 ± 0.52
Total Health Coaching Score		30.80 ± 2.04

Scoring system: 1 = Practice was not used, 2 = Practice was used partially, 3 = The practice was use consistently, 0 = NA.

**Table 6:** Mixed model ANOVA.

Variable	Group	Week 1	Week 2	Week 3	Week 4	Pre	Post	Interaction Effect *p*	Main Effect Time *p*	Main Effect Group *p*
Insole Usage (Hrs)	Public	12.76 ± 3.19	11.28 ± 9.73	9.52 ± 5.72	10.32 ± 6.48			0.408	0.389	0.723
	Private	12.34 ± 4.70	9.88 ± 2.77	12.54 ± 5.69	13.58 ± 6.75					
Number of Alerts	Public	35.94 ± 14.79	25.44 ± 16.79	22.24 ± 16.77	15.16 ± 12.83			0.442	0.047	0.438
	Private	19.16 ± 7.68	24.90 ± 25.74	13.50 ± 9.74	12.96 ± 15.29					
Percentage of Alert Non-Responses	Public	83.82 ± 3.78	64.66 ± 36.87	58.02 ± 36.42	65.20 ± 36.87			0.337	0.843	0.698
	Private	52.20 ± 17.16	63.90 ± 17.67	70.08 ± 20.14	59.60 ± 13.73					
Number of Alert Non-Responses	Public	30.00 ± 11.60	20.34 ± 13.54	17.60 ± 14.93	12.34 ± 10.82			0.236	0.035	0.318
	Private	11.42 ± 7.86	18.68 ± 23.33	9.00 ± 7.27	9.26 ± 12.93					
NAFF	Public					57.0 ± 10.77	61.60 ± 9.58	0.890	0.072	0.056
	Private					51.80 ± 3.11	56.70 ± 8.47			

Data are presented as mean ± SD.

**Table 7:** Questionnaire results *n* = 10.

Domain	Pre	Post	Mean Dif	Sig
UTAUT Means (0–4)				
Performance Expectancy	3.23 ± 0.79	2.38 ± 1.16	−0.85	0.060
Effort Expectancy	2.79 ± 0.63	2.84 ± 0.91	0.05	0.836
Attitude	3.23 ± 0.65	2.33 ± 1.04	−0.90	0.028
Social Influence	2.83 ± 0.50	2.63 ± 0.68	−0.20	0.405
Facilitating Conditions	3.13 ± 0.40	3.30 ± 0.51	0.17	0.209
Self-Efficacy	3.10 ± 0.57	3.60 ± 0.52	0.50	0.081
Anxiety	1.05 ± 1.19	0.43 ± 0.39	−0.62	0.182
Behavioural Intention	3.30 ± 0.88	1.37 ± 1.32	−1.93	0.008
Podiatrists UTAUT Means (0–4)				
Performance Expectancy	3.50 ± 0.00	2.63 ± 0.88	−0.87	0.395
Effort Expectancy	3.38 ± 0.88	3.38 ± 0.88	0.00	
Attitude	3.67 ± 0.47	3.00 ± 0.47	−0.67	
Social Influence	2.38 ± 0.18	2.13 ± 0.88	−0.25	0.705
Facilitating Conditions	3.13 ± 0.18	3.13 ± 0.18	0.00	
Self-Efficacy	3.17 ± 0.71	3.00 ± 0.94	−0.17	0.500
Anxiety	1.50 ± 0.71	0.75 ± 1.06	−0.75	0.205
Behavioural Intention	3.50 ± 0.71	1.00 ± 1.41	−2.50	0.344
NAFF Mean (0–78)	52.4 ± 8.81	56.70 ± 8.47	4.3	0.056
PIN Mean (1–5)				
ID1: Good circulation equals healthy feet	3.70 ± 0.79	3.43 ± 0.88	−0.27	0.093
ID2: Accurate interpretation of neuropathy	3.87 ± 0.57	4.23 ± 0.50	0.36	0.075
ID3: Foot ulcers would be painful	3.57 ± 0.93	3.03 ± 1.05	−0.54	0.168
C2: Blame of self or practitioner	2.60 ± 0.76	3.25 ± 0.59	0.65	0.014
C1: Physical causes of foot ulcers	3.18 ± 0.62	3.70 ± 0.63	0.52	0.023
TL: Understanding of ulcer onset	3.63 ± 0.92	4.00 ± 0.74	0.37	0.024
CC2: Practitioner control	2.77 ± 0.86	2.53 ± 0.95	−0.24	0.572
CC1: Efficaciousness of foot self-care	3.70 ± 0.76	4.14 ± 0.38	0.44	0.044
Cons: Consequences of neuropathy	4.35 ± 0.61	4.45 ± 0.42	0.10	0.653
EC1: Concern about possible consequences	4.33 ± 0.72	3.88 ± 0.92	0.45	0.134
EC2: Anger targeted towards practitioners	1.80 ± 1.23	1.95 ± 0.90	−0.15	0.752

Data are presented as mean ± SD.

**Table 8:** Podiatrist in-depth interview results.

Podiatrist In-Depth Theme	Participant Quote
Health Coaching Package	
Health coaching training session	*“I think it (health coaching training package) was sufficient information. I understood it and was able…to implement it through what I was shown… I felt very confident.” Pod1* *“I found the session engaging and quite informative and easy to follow in terms of developing and picking up some of those skills. Yeah, on reflecting from the sessions, I felt like I was confident that what I was teaching, in how I was interacting with the participants that they were up-taking those skills adequately.” Pod2* *“it (health coaching training) definitely…did help me to reflect on a few things more generally and broadly within my practice.” Pod2*
Written information	*“…before the consultations, I was able to read and remind myself exactly what needs to be communicated to the clients… and how to educate the clients on how to look after their feet as part of the whole programme.” Pod 1* *“I made sure I had most of the information (at) arm’s length, especially some of the Teach Back skills and health coaching aspects like the R.I.C.k. acronym… I had that…in my eyesight at most times to ensure I was covering through many of those points.” Pod2*
Health coaching impact on podiatrist and participant relationship	*“I think the relationship that I have with those clients has probably been enhanced as a result of that (health coaching intervention) as well.” Pod 1*
Improvements to health coaching intervention	*“…as far as I was concerned, I had done everything correctly (in issuing the insoles). I then found out subsequently that I had not calibrated them correctly… So, that may have been an issue as far as the initial calibration and the amount of alarms that some of the clients had. So, if there had been somebody there (supervising the initial consultation)…that would have changed the whole ball game as far as I was concerned.” Pod1*
Attitude	*“This is a technology (type)…I think will help in the long run. I don’t think this particular technology that I was involved in is what I could implement. But I think that this is a step in the right direction.” Pod 1* *“Any use of technology such as this to help patients monitor their feet better to reduce their likelihood of ulcer recurrence, or yeah, basically any diabetic foot complication at all would be certainly a worthwhile thing to explore.” Pod 2*
Performance Expectancy	*“I think that the modality itself has good possibilities to assist with the monitoring and looking after of people with neuropathy, particularly people that have a history of, or at obvious risk of plantar pressure lesions. Obviously, we had to advise our clients that there are areas dorsally or medial or lateral of (the) foot…(the smart insole) is not going to be monitoring so don’t be complacent about things.” Pod 1* *“Most of the interface being the smartwatch itself, the simple colour coding of the different pressure areas, the simplicity and the monitoring software and uploading the data. So, in that sense…I thought it was quite simple and the patients certainly seemed to understand and pick it up quite quickly.” Pod 2* *“…in terms of the physicality of it, we did have some issues in terms of the structural integrity and strength of a couple of devices, with some of them being damaged. So, in terms of…long term provision and use for clients in a normal everyday context…it would require a great deal of care and might not be suitable for people who remain fairly active.” Pod 2*
Self-Efficacy	*“…after a while you know exactly what you’re doing, you know exactly how it works, and…exactly how long it’s going to take unless there was a complication. And even then, you know, you’ve dealt with lots of complications eventually. So those complications…they start to become easier to deal with as well.” Pod 1*
Behavioural Intention	*At the moment, I would not adopt this technology…But I see it in the long run is something that would certainly be worthwhile, particularly…in public positions where there are…clients that would require this sort of thing who are always at risk.” Pod 1* *I’m not convinced in its current form or addition that it’s right to everyone, but not that any intervention would be right for every participant or patient. Pod 2* *“In the exact format that it is right now…I would have some reservations about it. Being able to customize the sensitivity a little bit better, may be of great benefit.” Pod 2*
Facilitating Conditions—cost	*“…with experience, I believe this could be easily done within a standard consultation, maybe a slightly longer consultation. Yes, unless something goes wrong, then all bets are off.” Pod 1* *“(Health coaching approach) probably didn’t make a substantial difference in terms of the overall consult time. You’re just probably spending time in different ways.” Pod1* *“From my experience in private practice in a regional area there is no market for it (smart insole) in private practice. I can’t see anybody paying (thousands) for this. Not my clients.” Pod 1* *“…if there was a possibility that this is going to be a technology that could be subsidised in some way for people that definitely do need this, then yes, it’s something that I would look at implementing.” Pod 1*
Other technology	*“if there was something else…that maybe also had thermal (sensors) that could tell you that there’s a hotspot rather than just a pressure spot…that’s going to communicate in a fashion that is easy for the person to monitor themselves without it being distracting …or overreacting all the time.” Pod 1*
Social Influence—participant related	
Anxiety	*“I think that they were a bit scared of the technology. Probably three out of the five would have been a bit concerned that this technology…was a little bit out of their understanding…they found it was very tricky to work with.” Pod 1*
Attitude	*“they (participants) were very keen and are very hopeful that this modality could be used to improve the lives of people with diabetes, peripheral neuropathy…So, it was something that they were very keen to try and make work.” Pod 1*
Self-Efficacy	*“They (participants) were…easy to contact, easy to be involved with, long standing clients that I know and have a relationship with, and of an intelligence that could handle this type of modality…And that I think is probably something that needs to be primarily understood…” Pod1* *“…the technology… wasn’t all that simple for them to get their head around, they had to understand quite a few things and they’re getting a lot of instructions and these are older people with less technology ability…” Pod 1*
Performance Expectancy	*“…my clients’ feedback particularly on their first review with me was that they found it clunky.” Pod1* *“…one of the feedback that came through was surprise at how much they’ve (participants) been just doing nothing standing still and getting pressure on one spot. But there were certain things that seemed like…the alarm went off for no particular reason.” Pod 1* *“From a consumer standpoint, I thought it (smart insole) was quite simple and the patients certainly seemed to understand and pick it up quite quickly. Pod 2*
Effort Expectancy	*“They also found that the alarms did go off a lot. That may have been associated with the calibration that we initially started with. On the first review, they came back…generally the feeling from just about everybody was that there were too many alarms…sometimes they didn’t know why they’re getting alarms…And, they found that did affect their lives…it affects their lives to the point they say, ‘I can’t use this technology for that reason if I have to stop every five to 10 min, or walk around for another two minutes’ …On the second week once…the calibration things (had) been sorted out…some of them seemed to settle down a bit (about the alarms) and they seem(ed) to be a bit more retrospect about it.’” Pod 1* *“A number participants were getting quite regular and frequent alarms despite…resetting them and trying to recalibrate them often and reconnect, doing all the typical points to try and reduce that frequency. Just to ensure that they were actually getting more meaningful information about it.” Pod 2*
Social Influence	*“It is quite a knob that they have to wear on the top of their shoes, which is not a major problem, but it is something that obviously stands out. Some people said that they felt that people noticed it…for most there wasn’t a major consideration, but I’m sure that there are particularly some women that would not choose to wear something that sticks out…it’s not attractive.” Pod1*

Identifier convention: Pod1 denotes the private arm podiatrist, Pod2 denotes the public arm podiatrist.

**Table 9:** Participant focus group results.

Focus Group Theme	Participant Quote
Health Coaching Intervention	
Knowledge—Impact on understanding of peripheral neuropathy, foot monitoring and care.	*“It (health coaching intervention) certainly made us more aware of our feet…and I would say, I’m washing now, I get the mirror and have a look to see if there’s any ulcers. Just a simple thing like that. I hadn’t thought of it. But I do it now.” P1a.* *“I knew nothing about my feet ‘till (the) podiatrist explained it to me.” P2a* *“Well, it was probably a revelation to the problems that can occur that I wasn’t aware of—that you had to keep checking your feet, because ulcers and stuff just crop up out of nowhere. I didn’t have a full understanding of the situation until I spoke to the podiatrist…It (health coaching session) was more specific…where before it was just general foot care. Now all of a sudden…the balls been thrown in our court and it’s saying, ‘you’ve got to dry between your toes, you’ve got to use cream, have a look for ulcers.’ So now I’m doing that where I wasn’t previously.” P3a* *“I think this time it was more in-depth. So before even if the other podiatrist would have told you something. It was just general. This time it was specific…It explained what could happen… if you don’t take really good care of your feet…It was really more informative than it had been before.” P4a* *“I’m fairly comfortable with where I’m at. It (health coaching in foot monitoring) probably reinforced what I already know.” P1b* *“I knew a fair bit of that (nature of peripheral neuropathy) before hand.” P2b* *“It (health coaching session) confirmed that what I was doing was correct. Because I had already had two situations where I had ulcers on my toes.” P2b* *“I found I was doing pretty well everything anyway, just as a matter of course.” P3b*
Knowledge—Smart insole usage explanation	*“…there was more than enough information to do the test (trial of insole) without any problems.” P1a* *“Yeah…there were no problems (understanding how to use the insole).” P3a* *“I didn’t have any problems of understanding what we had to do (to use the smart insole).” P1b”* *“Yeah, that (smart insole instruction) was fine.” P3b* *“The only thing I would do differently…about the training… would be to change (the insole into different shoes). I wore the same shoes because the innersole was in these shoes…rather than taking them out and putting them in other shoes.” P2b*
Knowledge—Smart insole written information	*“That (laminated response to alerts guide) was probably the most crucial! …I would have been lost without it.” P1a* *“(A quick reference trouble shooting guide)…would have been a good thing to have…troubleshooting charts would have been more helpful (than a booklet).” P3a* *“At that time, I really think that the booklet should have had more information in it and written more simply and explain it more the use and what could go wrong.” P4a* *“I read it (SurroSense Rx written information) when I went home, and just reinforced it.” P1b* *“I don’t reckon I even looked at it (written smart insole information). (The smart insoles) function(ed) properly…and what they (the podiatrist) said was going to happen happened.” P2b* *“It (instructions on resetting insole sensitivity) was augmented by the book.” P3b*
Podiatrist communication style during health coaching intervention	*“I couldn’t fault his (podiatrists’) approach…he’s made himself very available if there was any problems.” P1b* *“I felt, (the podiatrist went to) quite great lengths to make sure that what he was saying was got through and with this particular issue…to calm me down, and then another way to make sure that I was dealing with it. Well, that was well done.” P3b*
Performance Expectancy	*“I thought it would be a very good diagnostic tool for a podiatrist…But I don’t see how I’d use it personally. Unless, as you say you had ulcers and then you’re getting renewed ulcers…” P3a* *“…it’s got to be more user friendly, more discreet, and I felt that the sole was too thin, and needs to be more of a comfort sole built that you can put in any shoe quickly. And the recharging system needs to be a lot easier…” P3a* *“Just how specific do we know it is? Has it been proven that it works correctly?” P3a* *“I really felt it (smart insole) made me more aware of how I position my feet. It went off mostly when I was sitting down, and I thought how can it go off sitting down? And then I realized…when I sit down, I…put pressure on my toes…and so now I try not to do that. ” P4a* *“Well mine alerted a lot, but it didn’t show me anything… There were no surprises in what it alerted to. But I suppose the frustrating thing for me was that…it could alert and I could change things but…It (pressure) would go back, it goes back to that anyway.” P1b* *“I don’t mind about the alarms…(but) I found that watch cumbersome to wear…” P1b* *“I think it (smart insole) was beneficial for me because it alerted me and when it alerts me, I change it.” P2b* *“I think though there’s value there (in a smart insole). In my case it would show up (pressure)…before I was aware of…changes that…would cause long term problems.” P3b*
Trust	*“I believed that the pressure was on my feet…big toe area mainly come up…” P2a* *“it (smart insole) was good in the fact that it was actually showing you whether it was the heel or the toe. And it (pressure) was (caused by) the way I was…sitting.” P2b* *“You’d be just sitting there and they’d (smart insoles) go off, like you’ve got pressure on the foot. I’m thinking how can I when I’m sitting down? So, I never had a lot of confidence in what it was supplying.” P3a* *“I think that it didn’t work properly…because… the pressure…wasn’t there for that long…to give me an alarm…I was supposed to go and put my feet up. I didn’t do that.” P4a*
Effort Expectancy	*“Well, I thought it was all easy to use. I had no complicating factors. I did have to change mine into another pair of shoes on the second day. And that was easy…It was probably time consuming…You just couldn’t (get out of) bed and put shoes on…You had to…get connected to the world…so that was time consuming.” P1b* *“There was nothing difficult. It (smart insole) was annoying at times, but nothing difficult…connecting it was quite easy, provided that it stayed together (alluding to occasions when transmission pod came unglued).” P2b* *The daily routine (was easy). The charging of it (smart insole) and that sort of thing and connecting from it.” P3b*
Insole faults and technical issues	*“Well I tried resetting it (sensitivity)…(but) no matter what you do, that alarm will go off.” P1a* *“…one of them (smart insole) stopped completely…there was a slight crimping of the sole which had caused some connections in there to malfunction…” P3b* *“It’s not soldier proof.” P1a* *“Once in a while when you try to connect them and only one blue light comes on, and the other is red. And you have to start again, and again. And it does that three or four times you get really sick of it at the end.” P4a*
Intrusiveness	*“I initially started making notes on when it went off, what the actions were. And after an hour, I gave up. You know, I’d be using a ream of paper a day, just trying to keep on track with it.” P1a* *“…when you’re driving…your buzzer going off—there’s pressure on your feet. Well you just can’t pull up anywhere, get out and walk around for 20 min or 10 min.” P2a* *“At one stage I put them in my shooting shoes. I shoot competitively on the weekend. Well, I’m about to take a shot and this is going buzz, buzz, buzz putting me off. So, I had to stop wearing it on the weekend…I found that interfering with everyday routine and things that you were doing…” P3a* *“It was a nuisance because it went off. Every time I was trying to prepare dinner, standing up maybe, every time I was hanging out the clothes, and it went off in the car. So maybe it was too sensitive.” P4a*
Social Influence	*“The watch was a nuisance with the audible alarm. I was alone in the hotel having a beer and it went off and half the people were jumping over the bar thinking there was a bomb. It frightened the blooming life out of them. They didn’t know what it was.” P1a* *“It (smart insole) has to be more user friendly, so it doesn’t have components on top of me shoes like I’m walking out there like I’m Santa Claus with lights going off, and blokes going—‘what are those things?’…It’s got to be…more discreet.” P3a*
Facilitating Conditions—patient centred	
Footwear	*“Well, we weren’t given a time limit on it (how many hours a day to wear the smart insole), but (as) soon as I get home the first thing that goes is the shoes and the thongs come out, you know…(However) I thought, Oh, yeah, I better wear them until tonight anyway. So, I was over wearing what I normally would.” P3a*
Behavioural Intention to adopt smart insole	*“The technology definitely needs work. Particularly…with the pressurisation and the alarms, you know, they’re gonna decide what’s an alarm…” P1a* *“…it may do a great job in the future…with more development. But at the moment, I just don’t think it’s there” P3a* *“as it is now, I wouldn’t wear it. I found it too annoying. But if you could improve that…” P4a* *“I don’t think it (smart insole) would solve the problem that I’ve got. Maybe further down the track it might. But I need to do a lot of other things before I do something like that.” P1b* *“I wouldn’t want to be using it every day! Modify it to make it stronger, so it wouldn’t come apart. Having the top thing…smaller in size, so it isn’t so obvious, or not there at all (might change participant’s mind about adopting a smart insole in the future).” P2b* *“I think…there’s value there…if there is something going wrong in the feet then I’d like an early warning. And if that’s (smart insole) a way of doing it then fine.” P3b*
Attitude towards trialling other forms of electronic foot monitoring devices	*“Well personally, more broadly. I would love to see technology that can give you really good feedback… I think yes. In the future technology (participant would consider adoption)” P3a* *“Yeah, that (smart sock) could be good.” P3b* *“I can’t find a decent pair of socks—so (smart socks) would be good!” P2b*

Identifier convention: ‘P’ refers to participant, the numeral denotes the order in which each participant first spoke during the focus group, ‘a’ denotes the private arm focus group and ‘b’ denotes the public arm focus group.

## Data Availability

The datasets generated and analysed during the current study are available from the corresponding author on reasonable request.
